# Monoclonal Gammopathy as a Potential Cause of Pure Red Cell Aplasia: A Case Report and Literature Review

**DOI:** 10.7759/cureus.109134

**Published:** 2026-05-18

**Authors:** Pone De Weerdt, Ivan Van Riet, Karel Fostier

**Affiliations:** 1 Department of Hematology, Vrije Universiteit Brussel (VUB), Brussels, BEL; 2 Department of Hematology, Universitair Ziekenhuis Brussel (UZ Brussel), Brussels, BEL; 3 Hematology and Cell Therapy Laboratory, Universitair Ziekenhuis Brussel (UZ Brussel), Brussels, BEL; 4 Translational Oncology Research Center, Vrije Universiteit Brussel (VUB), Brussels, BEL; 5 Department of Hematology, AZORG, Aalst, BEL

**Keywords:** monoclonal gammopathy, monoclonal gammopathy of clinical significance, plasma cell dyscrasias, plasma cell-targeted therapy, pure red cell aplasia (prca)

## Abstract

Pure red cell aplasia (PRCA) secondary to plasma cell dyscrasias has been previously reported; however, these cases are rare and often lack clinical details. Here, we report the case of a 44-year-old female patient presenting with progressive normocytic anemia in the setting of an IgG-kappa monoclonal gammopathy, without meeting the diagnostic criteria for multiple myeloma or any other hematological malignancy. Although the initial diagnostic workup for PRCA was inconclusive, and despite a transient response to erythropoiesis-stimulating agents, the condition progressed to severe, transfusion-dependent anemia. PRCA was eventually confirmed and proved refractory to conventional immunosuppressive therapy. Remarkably, plasma cell-directed therapy achieved almost instantaneous normalization of erythropoiesis and rapid paraprotein clearance. This clinical response suggests a pathogenetic role for the monoclonal immunoglobulin. While we were unable to demonstrate an in vitro inhibitory effect of the patient’s serum on burst-forming unit-erythroid cultures, our observations, coupled with previously reported cases, support an antibody-mediated mechanism in the pathogenesis of this PRCA variant. We suggest that PRCA associated with monoclonal gammopathy may represent a rare, distinct clinical entity for which plasma cell-targeted therapy could be considered.

## Introduction

Pure red cell aplasia (PRCA) is a rare hematological disorder clinically characterized by isolated normocytic anemia and morphologically defined by the absence or near-absence of erythroid precursors on bone marrow examination. Acquired PRCA is classified into primary and secondary forms, with the latter characterized by its association with an identifiable underlying disorder. While the majority of acquired cases remain idiopathic, PRCA is strongly associated with specific conditions, most notably thymoma, large granular lymphocyte leukemia (LGLL), and parvovirus B19 infection [1].

Although its pathogenesis remains incompletely understood, the hallmark feature of PRCA, despite its clinical heterogeneity, is an autoimmune-mediated mechanism resulting in the maturation arrest of erythroid precursors. Most forms of secondary PRCA are thought to be T-cell mediated, involving selective destruction of erythroid precursors by autoreactive T- or natural killer cells. In contrast, a rarer subset is antibody-mediated, with immunoglobulin G playing a central pathogenic role [2]. This second mechanism becomes particularly relevant when exploring the role of plasma cell dyscrasias in the development of PRCA.

The literature establishes an association between plasma cell dyscrasias and PRCA [3], though evidence for this link is largely limited to case reports and small series, reflecting the rarity of PRCA. Increasingly, case reports have documented the co-occurrence of monoclonal gammopathy, associated with a small, indolent plasma cell clone, and PRCA. These observations suggest the existence of a distinct clinical variant of PRCA, potentially driven by an antibody-mediated immune mechanism [3-9]. Our case further supports the hypothesis that erythropoietic inhibition may be paraprotein-driven.

We further highlight the therapeutic dilemma encountered when PRCA becomes refractory to standard immunosuppressive therapies. A better understanding of the immunopathogenesis, particularly the mechanisms driving the selective targeting of erythroid progenitors, is critical for the development of more targeted and effective therapeutic strategies [2]. In the setting of monoclonal gammopathy-associated PRCA, plasma cells may be considered as a viable therapeutic target, given their putative role in producing pathogenic antibodies.

## Case presentation

A 44-year-old woman with no known comorbidities was referred to the hematology department for evaluation of moderate normocytic anemia. She reported several months of fatigue and exertional dyspnea. Her medical history was notable only for long-term oral contraceptive use and recent initiation of a multivitamin supplement. Physical examination revealed pallor, but vital signs were stable, functional status was preserved, and no dysmorphic features were noted.

Initial laboratory investigations confirmed normocytic, normochromic anemia (hemoglobin 9.3 g/dL, mean corpuscular volume 96.8 fL) with an inadequate reticulocyte response (reticulocyte production index 0.3). A peripheral blood smear showed poikilocytosis and ovalocytes. Serum vitamin B12, folate, and iron studies were within normal limits. Although the direct antiglobulin test (Coombs test) was positive, there was no biochemical evidence of hemolysis. Serum protein electrophoresis revealed an M-protein spike of 13.7 g/L (IgG-kappa isotype) and an altered serum free light-chain (kappa/lambda) ratio of 5.4. Urinary protein electrophoresis was also negative (Table 1).

**Table 1:** Laboratory data

Variable	On the first presentation	Reference range, adults
Hemoglobin (g/dl)	9.3	12–16
Hematocrit (%)	27.3	35-48
Mean corpuscular volume (fL)	96.8	80-100
Reticulocytes (x 10³/mm³)	23.1	20–100
White cell count (x 10³/mm³)	7.2	4–10
Differential count (%)		
Neutrophils	46	37.9-73
Lymphocytes	42.7	17.3-49.6
Monocytes	8.1	4.9-12.6
Eosinophils	3.7	≤7.3
Basophils	1.1	≤1.5
Platelet count (x 10³/mm³)	490	150-440
Iron (μg/dl)	92	37-145
Transferrin saturation (%)	35.4	15-45
Ferritin (μg/liter)	109	13-150
Vitamin B_12_ (ng/liter)	412	>197
Folic acid (μg/liter)	4.7	≥3.9
M-peak (g/liter)	13.7	-
IgG (g/liter)	17.5	7-16
IgM (g/liter)	0.61	0.4-2.3
IgA (g/liter)	1.99	0.7-4
Serum free kappa light chain (mg/liter)	49.3	3.3-19.4
Serum free lambda light chain (mg/liter)	9.2	5.7-26.3
Ratio of kappa to lambda free light chains	5.4	0.3-1.6

An initial bone marrow biopsy showed normocellular marrow with normal maturation of the three hematopoietic lineages, alongside a slight plasma cell excess (5-6%) with kappa light-chain restriction. PET-CT imaging showed no bone lesions, mediastinal masses, or other evidence of malignancy. The patient did not fulfill the diagnostic criteria for multiple myeloma, as defined by the International Myeloma Working Group (IMWG) [10].

Shortly thereafter, the patient became transfusion-dependent. A repeat bone marrow biopsy revealed mild dyserythropoiesis and occasional ring sideroblasts. While a slight plasma cell excess persisted (5-6%), it remained stable without progression. These morphological findings in the erythroid lineage initially suggested a diagnosis of myelodysplastic syndrome (MDS); however, karyotype analysis and myeloid next-generation sequencing failed to show evidence of clonal hematopoiesis. Given a presumptive diagnosis of low-risk MDS, a trial with an erythropoiesis-stimulating agent was initiated, which maintained transfusion independence for two years.

**Figure 1:**
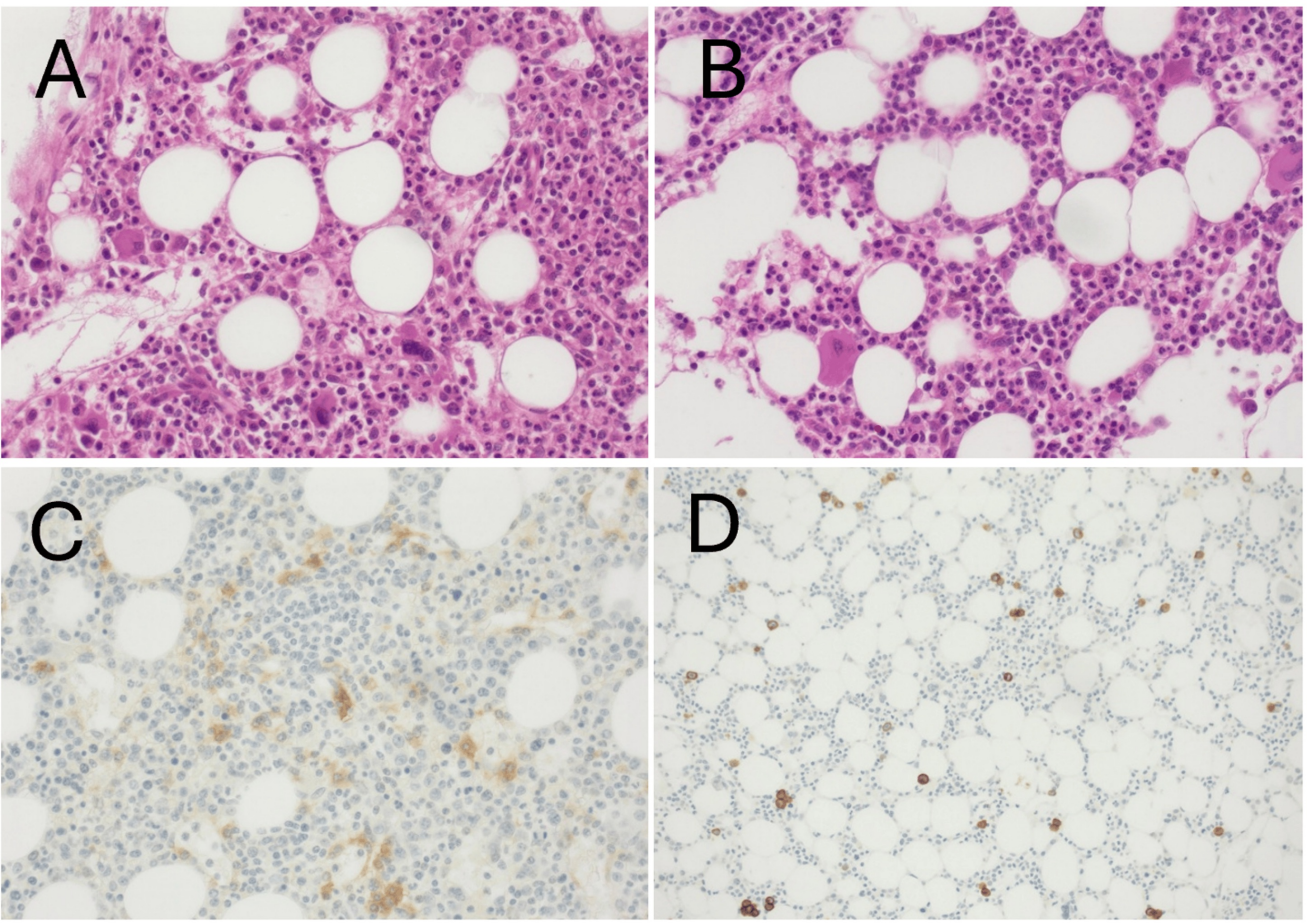
Bone marrow trephine biopsy Panels A and B (200x, H&E) show a normocellular bone marrow with preserved myeloid lineages and megakaryocytes, but with a reduced number of erythroid islands. Panel C (100x, CD78 immunohistochemical stain) demonstrates a markedly reduced number of immature erythroid cells. Panel D (40x, CD138 immunohistochemical stain) shows a slight excess of plasma cells.

Subsequently, the patient re-presented with severe anemia and profound reticulocytopenia. A third bone marrow biopsy demonstrated a near-complete absence of erythroid precursors, compatible with a diagnosis of PRCA (Figure 1). Common secondary causes of PRCA were excluded.

The patient initially demonstrated an encouraging response to first-line high-dose corticosteroid therapy (64 mg of methylprednisolone daily for three weeks, followed by a gradual taper), but experienced a relapse during the tapering phase at three months. Escalation to combination therapy with corticosteroids and cyclosporine A (CSA) failed to achieve a response.

**Figure 2:**
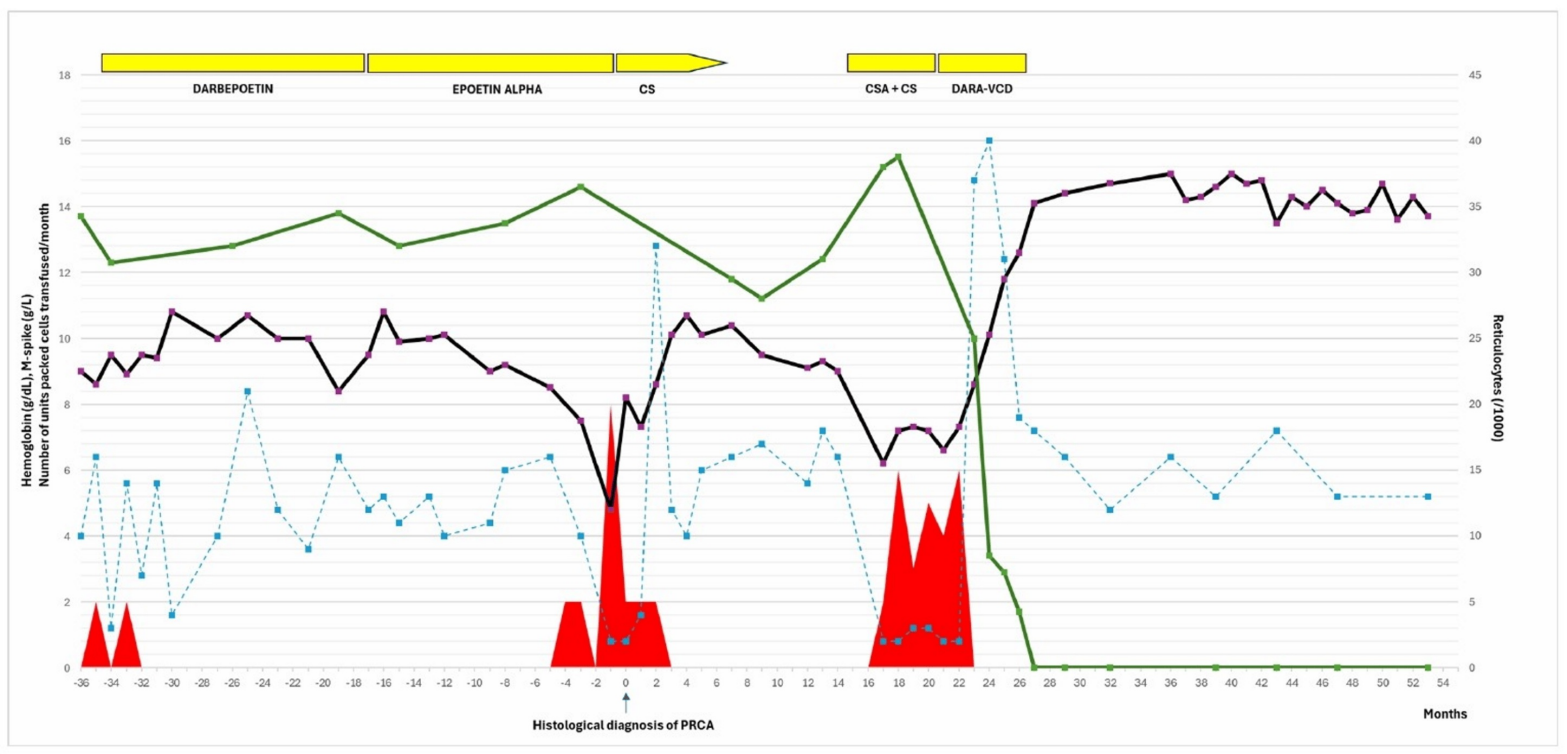
Evolution of biological parameters during disease progression and treatment Time course (in months) before and after histological diagnosis of PRCA (time 0, indicated by arrow) showing: hemoglobin concentration (g/dL, black line), M-spike (g/L, green line), absolute reticulocyte count (per 1000 red blood cells, dashed blue line) and monthly packed red blood cell transfusions (units, filled red line). Also depicted is the time course of the different therapies administered. CS (corticosteroids with dose taper), CSA + CS (ciclosporin A with corticosteroids), Dara-VCD (daratumumab-bortezomib-cyclophosphamide-dexamethasone). PRCA, pure red cell aplasia

Hypothesizing that the monoclonal gammopathy was the underlying driver of the PRCA, we first attempted to demonstrate in vitro that the patient’s serum IgG inhibits erythroid precursor maturation in bone marrow cultures (cf. description of CFU-G/M/GM and erythroid burst‑forming unit (BFU-E) assays). Despite being unable to confirm this inhibitory effect in vitro, plasma cell-directed therapy was initiated. The patient received six monthly cycles of daratumumab, bortezomib, cyclophosphamide, and dexamethasone (Dara-VCD). Shortly after initiation, a rapid and sustained clinical response was observed, characterized by normalization of hemoglobin levels, a robust increase in reticulocyte count, and complete suppression of the paraprotein (Figure 2).

Description of CFU-G/M/GM and BFU-E assays

Mononuclear cells (MNCs) were isolated from a pretreatment bone marrow sample by Ficoll density-gradient separation. MNCs were plated at a density of 50,000 cells per Petri dish in MethoCult GFH4434 growth medium (STEMCELL Technologies, Saint‑Égrève, France), supplemented with IL‑3, GM‑CSF, and stem cell factor, with or without the addition of erythropoietin (EPO). In parallel, MNCs were cultured in the same medium supplemented with EPO in the presence of patient serum. Cultures were incubated for 14 days at 37 °C in a humidified atmosphere containing 5% CO₂. Cultures without added EPO were scored for myeloid colony-forming units (CFU‑GM/G/M), defined by the presence of granulocyte and/or monocyte colonies. Cultures supplemented with EPO were scored for BFU‑E, both in the presence of patient serum and in Iscove’s medium used as a control.

The bone marrow sample demonstrated normal hematopoietic stem cell activity. Colony numbers for both the myeloid CFU assay (141 colonies per 10⁵ MNCs) and the BFU‑E assay (156 colonies per 10⁵ MNCs) were within the normal reference ranges (70-220 and 50-270 colonies per 10⁵ MNCs, respectively). The addition of autologous serum did not result in significant inhibition of BFU‑E growth (154 colonies per 10⁵ MNCs).

Review of the literature

To further contextualize our findings, we conducted a review of PubMed-indexed literature. Our initial search identified 17 observational studies documenting an association between plasma cell dyscrasias and PRCA. To maintain the focus on 'erythroid significance' rather than malignancy-associated marrow failure, we excluded patients meeting diagnostic criteria for smoldering or symptomatic multiple myeloma and Waldenström macroglobulinemia. We describe the characteristics of these patients, as outlined in Table 2.

**Table 2:** Overview of published case reports of monoclonal gammopathy-associated pure red cell aplasia †: Treatment was discontinued due to intolerance. ‡: Treatment was continued as maintenance therapy. *: Two lines of unspecified immunosuppressive therapy were administered. NA, not available; LGLL, large granular lymphocyte leukemia; GCA, giant cell arteritis; SLE, systemic lupus erythematosus; rhEPO, recombinant human erythropoietin; CS, corticosteroids; CSA, cyclosporine A; Sir, sirolimus; Dara-VCD, daratumumab – bortezomib – cyclophosphamide – dexamethasone; VRd, bortezomib – lenalidomide – dexamethasone; Vd, bortezomib – dexamethasone; Rd, lenalidomide – dexamethasone; R, lenalidomide; Kd, carfilzomib – dexamethasone; Id, ixazomib-dexamethasone; Isa-Pd, isatuximab – pomalidomide – dexamethasone; IVIg, intravenous immunoglobulins; CR, complete remission; PR, partial remission; FLC, free light chain.

	Patient	Sex	Age (years)	Comorbidity	Hemoglobin level (g/dl)	Monoclonal protein (g/L)	FLC ratio	% monoclonal plasma cells in bone marrow	Prior therapies	Plasma cell-targeted therapy (duration in months)	Outcome (follow-up in months)
Index patient	1	Female	44		9,3	IgG κ (13.7)	5.4	5-6	rhEPO	Dara-VCD (6)	CR (30)
									CS		
									CSA		
Huang et al. (2025)[4]	2	Female	58		4.1	IgG κ (18.6)	NA	2	CSA	Vd (3)	CR (24)
									CS	VRd (4)	
									Sir	R †	
	3	Female	51	LGLL	4.4	IgA κ (2.4)	NA	0.5	CSA	VRd (7)	CR (16)
									Sir	R (6)	
	4	Male	69		4.9	IgA κ (4.3)	NA	1.5	CSA	Rd ‡	PR ‡
									Sir		
Khan et al. (2024)[5]	5	Male	65	GCA	8.9	IgG λ (8.0)	NA	5	NA	NA	NA
Michel et al. (2024)[6]	6	Male	44		9.0	IgG λ (6.0)	2.3	5	CSA	Dara-Vd (8)	CR (36)
										Kd (6)	
										Id (2)	
										Isa-Pd †	
Zhang et al. (2021)[7]	7	Male	45		4.4	IgG κ (3.1)	NA	2	CSA	Vd (3)	CR (12)
									CS		
									rhEPO		
									IVIg		
	8	Female	30	SLE	5.0	IgG κ(8.6)	NA	2	CS	Vd (3)	CR (8)
Eziokwu et al. (2019) [8]	9	Female	61		8.9	IgG (4.4)	NA	5	IVIg	Vd (5)	CR (NA)
									CSA		
Gu et al. (2017) [9]	10	Male	55		NA	IgG λ (NA)	NA	7	NA	NA	NA
Korde et al. (2016) [3]	11	Male	67		8.0	IgG λ (6.0)	0.48	5-9	NA	Rd (NA)	CR (NA)
										R (NA)	
	12	Female	51		6.4	NA (9.0)	12.4	10	*	-	NA

This filtered cohort of patients encompasses 12 patients (six female patients, six male patients), including the index patient, aged 30 to 69 years. Most patients had no significant comorbidities; however, three had been diagnosed with LGLL (patient 3), giant cell arteritis (patient 5), and systemic lupus erythematosus (patient 8), prior to the onset of PRCA. At presentation, hemoglobin levels ranged from 4.1 to 9.3 g/dL, with uniformly low reticulocyte counts. The monoclonal protein was predominantly IgG (kappa or lambda), with a minor subset demonstrating an IgA (kappa or lambda) paraprotein. Monoclonal protein concentrations spanned a broad range (2.4-18.6 g/L), accompanied by variable bone marrow plasma cell infiltration.

Prior to plasma cell-targeted therapies, nine of the reported patients had received between one and four prior lines of treatment, most commonly including CSA. Three classes of plasma-cell targeted therapies were used: proteasome inhibitors, anti-CD38 monoclonal antibodies, and immunomodulators. The most frequently used regimens were bortezomib-dexamethasone (Vd) or bortezomib-lenalidomide-dexamethasone (VRd). In two patients (patients 2 and 6), two and four lines of plasma cell-targeted therapy, respectively, were required to achieve a response. Treatment durations varied, typically spanning two to eight months, with two patients continuing therapy on a prolonged basis (patients 2 and 6).

When reported, initial clinical outcomes were generally favorable, with predominantly rapid reticulocyte responses following initiation of plasma cell-targeted therapy and achievement of transfusion independence. Follow-up periods ranged from 6 to 36 months, though data were not available for all patients. No data on relapses were reported.

## Discussion

This case underscores several key points for the contemporary management of PRCA.

First, PRCA is a heterogeneous disorder, as reflected in its variable clinical course, associated conditions, and management strategies. Diagnostic delay, often accompanied by increased morbidity and impaired quality of life, remains a significant clinical challenge. This is frequently attributable to the insidious onset of the disease and the reliance on characteristic bone marrow morphological features, which may be subtle or absent during early stages. 

Second, there has been an increase in case reports describing a link between plasma cell dyscrasias and PRCA, the largest series of which was reported by Korde et al. in 2016 [3]. Current diagnostic algorithms recommend serum protein electrophoresis, immunofixation, and serum free light-chain analysis during the evaluation of PRCA to identify potential underlying B-cell disorders [11,12]. However, recognition of PRCA as a distinct clinical and pathological entity in the context of plasma cell dyscrasias may be challenging, given that anemia is a hallmark feature of common plasma cell disorders.

Nevertheless, this case adds to a growing body of reports [3-9] describing the co-occurrence of monoclonal gammopathy and PRCA in the absence of an overt hematologic neoplasm (Table 2). We suggest that monoclonal gammopathy may act as a potential driver of erythroid inhibition in PRCA. This hypothesis is supported by several observations.

Clinically, erythroid recovery is observed following a reduction or clearance of the paraprotein in most reported cases. In addition, conventional immunosuppressive therapies often prove ineffective, whereas targeted treatment against the plasma cell clone achieves high response rates, as detailed below. Immunologically, the majority of implicated M-proteins are of the IgG isotype (Table 2), and IgG-mediated inhibition of erythropoiesis is a well-established mechanism in both primary (autoimmune) PRCA and transient erythroblastopenia of childhood, considered analogous to PRCA in children [13,14].

To explore this potential mechanism in our patient, we conducted an assay that demonstrated the patient's anemia could not be attributed to intrinsic bone marrow failure. Instead, the inhibition of erythropoiesis appeared to be driven by an extrinsic factor. However, we were unable to directly attribute this inhibition to the paraprotein (cf. description of CFU-G/M/GM and BFU-E assays). One possible limitation is that the use of whole serum may have diluted the paraprotein to concentrations insufficient to demonstrate inhibition in vitro. Furthermore, the relevant antigen may be expressed mainly in later erythroid progenitors rather than in BFU-E cells [14]. Alternatively, the pathological mechanism may be more complex than the action of an antibody against an antigen expressed on erythroid precursors.

However, our findings align with the broader concept that monoclonal gammopathy itself, regardless of tumor burden, can drive systemic disease. This was formally recognized in 2018 with the introduction of the term "monoclonal gammopathy of clinical significance" [15]. While further research is needed to clarify the role of monoclonal gammopathy in PRCA, its presence in PRCA should not be dismissed as an "innocent bystander." In this context, the term “monoclonal gammopathy of erythroid significance” could be considered, by analogy with monoclonal gammopathy of renal significance or monoclonal gammopathy of neurological significance.

Third, PRCA refractory to standard immunosuppressive therapy remains a therapeutic challenge. The most commonly employed first-line treatments for PRCA are CSA, corticosteroids, or a combination of both, with reported response rates ranging from 60% to 90% [16]. However, it is well-established that these response rates vary significantly depending on the underlying etiology. Consequently, the management is evolving toward a more tailored approach that recognizes distinct variants arising from different immunopathogenic mechanisms [2,11,12].

Within this context, literature on plasma cell dyscrasia-associated PRCA frequently reports resistance to conventional therapeutic regimens. Growing evidence suggests that anti-plasma cell therapy offers significant clinical benefit in this variant [3-9]. Proteasome inhibitors, with or without immunomodulatory agents, are the most commonly reported interventions (Table 2), often leading to significant erythropoietic recovery.

In our patient, the Dara-VCD regimen (daratumumab, bortezomib, cyclophosphamide, and dexamethasone) led to rapid hematological recovery following prompt paraprotein clearance. While this is an off-label indication, this strategy was clinically decisive. At two and a half years post-initiation, the patient remains in clinical remission after completing the six-month fixed-duration therapy, which was well-tolerated.

While the benefit-risk balance of anti-plasma cell therapy must be carefully weighed in non-malignant gammopathies, evidence supporting its use in PRCA is increasing. Recent studies have also explored these therapies in primary autoimmune PRCA and ABO-incompatible allogeneic transplant-associated PRCA, showing promising efficacy, potentially indicating a shared underlying mechanism, and acceptable toxicity profiles [17,18].

A limitation of this report is that plasma cell-targeted therapy has broad immunomodulatory effects, which might contribute to its efficacy independent of the monoclonal gammopathy. However, the strict temporal association and biologic plausibility strongly suggest that targeting the plasma cell clone, and thereby suppressing paraprotein production, was central to the restoration of erythropoiesis in our patient. Future research should help clarify whether PRCA associated with monoclonal gammopathy represents a distinct entity and define the role of plasma cell-targeted therapy in its management.

## Conclusions

PRCA remains a heterogeneous disorder that presents significant diagnostic and therapeutic challenges. We describe a distinct variant associated with monoclonal gammopathy, likely driven by a paraprotein-mediated, antibody-dependent mechanism. In our patient, targeting the underlying plasma cell clone resulted in a rapid and durable hematologic response. Our findings underscore the importance of tailoring therapy to the specific immunopathogenesis of PRCA. They also support exploring anti-plasma cell therapy as an alternative for cases linked to monoclonal gammopathy that do not respond to standard treatment regimens.
